# California Autism Prevalence Trends from 1931 to 2014 and Comparison to National ASD Data from IDEA and ADDM

**DOI:** 10.1007/s10803-018-3670-2

**Published:** 2018-07-05

**Authors:** Cynthia Nevison, Mark Blaxill, Walter Zahorodny

**Affiliations:** 1 Institute for Alpine and Arctic Research, University of Colorado, Campus Box 450, Boulder, CO 80309-0450 USA; 2 Health Choice, Cambridge, MA USA; 3 Rutgers University – New Jersey Medical School, Newark, NJ USA

**Keywords:** Autistic disorder, Autism spectrum disorder, ASD prevalence, Time trends, ADDM, IDEA, CDDS

## Abstract

Time trends in U.S. autism prevalence from three ongoing datasets [Individuals with Disabilities Education Act, Autism and Developmental Disabilities Monitoring Network, and California Department of Developmental Services (CDDS)] are calculated using two different methods: (1) constant-age tracking of 8 year-olds and (2) age-resolved snapshots. The data are consistent across methods in showing a strong upward trend over time. The prevalence of autism in the CDDS dataset, the longest of the three data records, increased from 0.001% in the cohort born in 1931 to 1.2% among 5 year-olds born in 2012. This increase began around ~ 1940 at a rate that has gradually accelerated over time, including notable change points around birth years 1980, 1990 and, most recently, 2007.

**Electronic supplementary material:**

The online version of this article (10.1007/s10803-018-3670-2) contains supplementary material, which is available to authorized users.

## Introduction

Autism was first described in the 1940s as a childhood psychiatric disorder characterized by early expressed impairment in social interaction and communication and repetitive or circumscribed interests or behavior (Kanner 1943). Kanner’s original term for the condition was *early infantile autism* or *infantile autism*. While originally attributed by some to bad parenting (i.e., “refrigerator mothers”) (Bettelheim 1967), today autism is widely recognized as a complex developmental disorder triggered by environmental factors acting on a genetically-susceptible population, in which inflammation may interfere with early brain synapse formation and pruning (Pardo et al. 2005; Goines and Ashwood 2012; Bilbo et al. 2015). Autism frequently co-occurs with other neurological and behavioral conditions (Van Der Meer et al. 2012) and is often accompanied by elevated levels of cellular oxidative stress, mitochondrial dysfunction, and/or immune and gastrointestinal disorder (James et al. 2009; Chaidez et al. 2013; Frye and James 2014).

Autism is diagnosed by confirmation of behaviors by experts, as there are no valid biomarkers or determinative tests. Autism diagnostic criteria were formalized for the first time in the 3rd Edition of the American Psychiatric Association’s (APA) *Diagnostic and Statistical Manual of Mental Disorders* (*DSM-III*) (APA 1980) to clarify the difference between *infantile autism* and childhood schizophrenia. In a subsequent revision to *DSM-IV*, published in 1994 (APA 1994), three autism subtypes were described: *autistic disorder* (*AD*), *pervasive developmental disorder-not otherwise specified* (*PDD-NOS*) and *Asperger’s syndrome*. These latter subtypes represent milder, variant forms, while *AD* is the most severe expression of autism. By definition, *AD* is in place by age 3, although it typically is not diagnosed until a median age of 4 years (MacFarlane and Kanaya 2009; CDC 2016).

*DSM-5*, published in November, 2013, formally defined the term *autism spectrum disorder* (*ASD*), which encompasses but no longer distinguishes between *AD, PDD-NOS* and *Asperger’s syndrome*, based on the rationale that the clinical distinction between the subtypes is not well defined (APA 2013). Indeed the CDC Autism and Developmental Disabilities Monitoring (ADDM) Network reports a wide range of variability among individual states in the proportion of ASD cases assigned to each subtype. Among 8 year-olds surveyed across 11 states in 2012, AD accounted for 26–74% (46% on average) of ASD cases, PDD-NOS accounted for 15–58% (44% on average) and Asperger’s for 2–19% (10% on average) (CDC 2016). Under DSM-5, in place of the old subtypes, clinicians rate the severity of deficits in two principal domains of (1) social communication and interaction and (2) restrictive and repetitive patterns of behavior (Gibbs et al. 2012; Volkmar and Reichow 2013).

Epidemiologic estimates of autism prevalence in the United States were in the range of 1 in 2500 prior to 1985, but increased to 1/150 among 8 year-olds born in 1992 and again to 1/68 for 8 year-olds born in 2002 (McDonald and Paul 2010; CDC 2016). Despite the rapid and broad rise in the reported ASD prevalence, a number of analyses have concluded that much of the apparent rise may not reflect a real increase in ASD cases. Rather, these studies have argued that diagnostic substitution for intellectual disability, the expansion of diagnostic criteria, and improved awareness of the condition have played an important role in explaining the increase in ASD (Croen et al. 2002; Gurney et al. 2003; Fombonne 2009; Keyes et al. 2012; Polyak et al. 2015). The epidemiologic literature also has suggested that the large variation in reported ASD prevalence from different geographic regions (e.g., the more than threefold differences among states in ADDM surveys) indicates a level of inconsistency in the data that precludes drawing conclusions about time trends (Fombonne 2009).

A recent analysis of data from the U.S. Department of Education Individuals with Disabilities Education Act (IDEA) offered a different view, finding that the large majority (75–80%) of the increase in ASD prevalence, over the period from 1988 to 2002, was due to a true increase in the condition rather than to better and expanded diagnosis (Nevison 2014). That investigation, which focused on IDEA data, compared two independent methods for calculating the trend slope of ASD prevalence versus birth year and found that both methods gave largely consistent results. The methods involved (1) tracking prevalence at a constant age over multiple, successive years of reports, and (2) examining an age-resolved snapshot from the most recent individual year’s report. The conceptual distinction between methods 1 and 2 was also important in demonstrating that diagnostic substitution for intellectual disability is an unsatisfactory explanation for the rise in ASD in most states, including California (Blaxill et al. 2003; Croen and Grether 2003; Nevison and Blaxill 2017).

The “constant-age tracking” method is the approach adopted by the ADDM network, which tracks ASD prevalence among 8 year-olds in successive biannual reports (CDC 2016). Two other data systems compile successive annual reports with separate counts for each age from early childhood to adulthood. These include IDEA and the California Department of Developmental Services (CDDS). The successive annual reports from these networks not only allow for constant-age tracking of any age cohort, but also effectively provide the opportunity to compute a prevalence snapshot, resolved by age, for any given report year. Using simple algebra, a prevalence versus birth year curve can be constructed from any of the individual age-resolved reports, thereby providing an independent, alternative approach to constant-age tracking for estimating the time trend in autism prevalence.

This alternative approach is referred to here as the “age-resolved snapshot” method. A key advantage is that the time trend derived from an age-resolved snapshot is substantially insulated from the biasing influences of better and/or expanded diagnosis, since these influences potentially may affect all age cohorts in the snapshot equally, provided the cohorts are old enough to be full ascertained. The age of full ascertainment for ASD commonly (although perhaps inappropriately) has been assumed to be about 8 (CDC 2016). Thus, in principle if ASD is truly a constant prevalence condition, a snapshot-based prevalence versus birth year plot, beginning around age 8 and extending back in time to older birth cohorts, should be a flat line with a slope of 0. In practice, children of different ages are not necessarily equally likely to be evaluated for ASD in a given year, and adults are even less likely to be evaluated. However, the IDEA law includes the Child Find mandate, which requires that all U.S. school districts locate and evaluate all children with disabilities from birth through age 21, suggesting there is an ongoing legal mandate to identify children with ASD throughout their school years (Wright and Wright 2007).

Earlier studies using “age-period-cohort” approaches to understand the interaction of age, cohort and report year effects (Gurney et al. 2003; Newschaffer et al. 2007; Keyes et al. 2012) have employed some of the same concepts involved in the age-resolved snapshot versus constant-age tracking method. Those studies have focused largely on following specific birth cohorts as they age and have noted a tendency toward an ongoing increase in prevalence within a given cohort well beyond age 8, as well as an overall increase in prevalence among younger versus older birth cohorts. However, the previous studies generally have not plotted or defined the time trend in autism per se, visualized as a simple graph of prevalence versus birth year.

In this paper, we apply the age-resolved snapshot and constant-age tracking method to a set of 13 CDDS annual reports, which date as far back as birth year 1931 and extend through birth year 2014. The *DSM-IV* definitions were used for most of these reports, although the two most recent reports were transitioning to *DSM-5*. For the older data, we use the *DSM-5* term ASD to refer to the sum of AD, PDD-NOS and Asperger’s syndrome. We compare the CDDS trends to trends in ASD from the California IDEA dataset. In addition, we calculate the IDEA ASD trend slopes in the 15 states surveyed by the ADDM Network, which allows for comparison of 8 year-old constant-age tracking trends between IDEA and ADDM. Our primary goal is to quantify and characterize the time trend in U.S. autism prevalence as well as possible using the best available data. Two secondary goals are (1) to test the hypothesis that the trend slopes derived from the most recently available age-resolved snapshots for the CDDS and IDEA datasets are significantly greater than zero (i.e., not flat lines), and (2) to examine reasons for the large variations in reported ASD prevalence among different states and data networks.

## Methods

### Autism Prevalence Data

Table 1 summarizes the three main sources of ASD prevalence data used in this paper. Each dataset is described in greater detail below and the complete datasets are provided in Supplementary Files S1–4. Since all relevant information had been de-identified prior to our activities and since the datasets were aggregated by age at the state level, this project did not require institutional review and approval.

**Table 1:** Summary of ASD datasets

Dataset	CDDS	IDEA	ADDM
Regions covered	California	All 50 states + D.C.	Selected counties in up to 15 states, varying by report
Age of autism counts	3–83	3–21	8
Denominator used to estimate prevalence	California birth data 1931–2014	NCES public school populations K-12 (age 5–17)	U.S. Census data
ASD types included	Code 1 autism (mainly AD)	Varies by state. Some may include only AD, others some or all ASD types	All ASD types, including Asperger’s syndrome
Report years	1997–2006, annually, 2014, 2016, 2017	1991–2011, annually	2000–2012, biannually
Sponsoring agency	Calif. Department of Developmental Services	U.S. Department of Education	U.S. Centers for Disease Control and Prevention
Supplementary data files	S1	S2, S3	S4

### California Department of Developmental Services (CDDS)

CDDS provides services to eligible individuals living in California who meet the DSM diagnostic criteria for autism. To qualify for CDDS services, these individuals also must have a level of impairment that rises to the level of a “developmental disability,” where the latter is defined as a non-physical, substantial disability that is expected to continue indefinitely (Autism Society San Francisco Bay Area 2015). Historically, the CDDS screening system has reserved the name “autism” for “full syndrome” cases, which have a modal age of 3 at diagnosis (Fountain and Bearman 2011) and are almost always diagnosed with AD. Official CDDS publications have focused on this more severely affected population (CDDS 2003). Milder subtypes such as Asperger’s syndrome and PDD-NOS have not been eligible for services unless they have another qualifying disability (Fountain and Bearman 2011). In addition to an autism diagnosis, individuals applying for CDDS services must demonstrate significant functional disability in 3 out of 7 life challenges, which include self-care, language, learning, mobility, self-direction, capacity for independent living and economic self-sufficiency (Autism Society San Francisco Bay Area 2015).

For the current study, CDDS autism counts were obtained as a set of 10 consecutive annual reports for 1997–2006 and 3 additional reports for 2014, 2016 and 2017. Each of the annual reports provides an age-resolved snapshot for that year of the number of individuals receiving services for autism. The counts are listed back to birth year 1970 for the 2016 report and back to birth year 1931 for all other reports. The 2014 snapshot was obtained from a published report (Autism Society San Francisco Bay Area 2015) while all the other reports were obtained through direct requests to CDDS. The data include only individuals who are “active” in the system and are classified under Code 1 on their Client Development Evaluation Report (CDER).

The definition of Code 1 has changed several times over the years within the CDDS system (http://www.dds.ca.gov/CDER/Index.cfm). In May, 2007 (but not implemented until November 2008), Code 1 was revised from its historic name, “autism, full syndrome,” to “autistic disorder (AD).” The two terms are similar (CDDS 2003), but the latter was adopted when CDDS began using separate codes 3 and 4 for Asperger’s syndrome and PDD-NOS, respectively, under the DSM-IV nomenclature. In November 2014, Code 1 was revised again to “autism spectrum disorder” as CDDS began shifting to the DSM-5 framework. The revised definitions initially apply only to new cases entering the system, while older cases retain their original classifications. However, most consumers are on an annual diagnosis update schedule (a minority are on a triannual schedule) with the result that 98% are transitioned within 2 years and 99% within 3 years to the new codes (Paul Choate, personal comm. 1/30/18). The chronology is such that the original definition of Code 1 (autism, full syndrome) applies to the 1997–2006 snapshots, the DSM-IV definition “autistic disorder” applies to the 2014 snapshot, and the new DSM-5 definition of Code 1 applies to the 2016 and 2017 snapshots.

The Code 1 autism counts were converted to prevalence in % for birth years 1970–2014, using California live birth data as denominators (http://www.dof.ca.gov/research/demographic/reports/projections/births/), consistent with the methodology used by CDDS (2003). The 1931–1969 counts were converted into prevalence using estimated California births from http://www.dof.ca.gov/Forecasting/Demographics/Estimates/E-7/. These birth estimates extend to the present day and, in the overlapping range, agree well with the 1970–2014 live birth estimates, to within a standard deviation of ± 9000. The full set of 13 age-resolved annual reports (1997–2006, 2014, 2016 and 2017) is available in Supplementary File S1.

### Individuals with Disabilities Education Act (IDEA)

The Individuals with Disabilities Education Act (IDEA) requires the collection of special education enrollment counts for 13 specific disability categories. IDEA is federally mandated and regulated under the U.S. Department of Education, but allows individual states discretion in determining special education categories, without reference to DSM or other diagnostic criteria. Rather, the determination of whether a student qualifies for autism services is made by district-level professionals in concert with the student’s parents and teachers (MacFarlane and Kanaya 2009).

ASD counts were obtained from the IDEA database for each of the 50 United States (http://www.ideadata.org). For report years through 2011, ASD counts for children age 6 through 17 are available in age-resolved annual reports beginning in 1991, while counts for 5 year-olds are available beginning in the 2000 report. ASD prevalence was calculated by dividing the IDEA counts by total statewide public school populations from the National Center for Education Statistics (NCES) (http://nces.ed.gov/ccd/elsi/). The NCES data are resolved by grade from kindergarten (age 5) to 12th grade (age 17) and are available in annual reports from 1991 to 2011. While additional IDEA reports have been published beyond 2011, from the 2012 report onward the findings were reformulated such that ASD counts are no longer available in age-resolved format, but rather are aggregated into broad age categories. These more recent reports do not provide the age-resolved data that our methodology requires.

The full datasets of IDEA ASD counts and computed IDEA/NCES ASD prevalence are available in Supplementary Files S2 and S3, respectively. Both datasets are presented for all 50 United States, although this paper focuses on the 15 states in which ADDM data are available, and on California, where CDDS data are available. We also compute the overall U.S. ASD prevalence by summing all (non-blank) data from all 50 states plus Washington, D.C. and dividing by the sum of the NCES public school populations in those states. In the early 1990s, only about half of states provided ASD counts, but data are available from at least 48 states for every year thereafter.

### Autism and Developmental Disabilities Monitoring (ADDM) Network

The Autism and Developmental Disabilities Monitoring (ADDM) Network is a surveillance system conducted in selected regions of the United States that was established by the Centers for Disease Control (CDC) in 2000 to provide estimates of autism prevalence among 8 year-old children. Reports are available biannually for birth years from 1992 to 2004, for a total of 7 reports (CDC 2007a, b, 2009a, b, 2012, 2014, 2016). ADDM ASD cases are determined by systematic review and abstraction of information contained in existing evaluations conducted for developmental health and special education purposes, followed by independent scoring and analysis by experienced clinicians to determine which children satisfy the DSM-IV-based definitions of ASD. (Note, in some states ADDM researchers have access only to health records and not education records.) ADDM uses U.S. Census-based data for the age cohort denominators needed to compute prevalence. ADDM data cover all ASD subtypes, including AD, PDD-NOS and Asperger’s disorder. While ADDM, over the lifetime of the Network, covers parts of 15 different states, the states surveyed are not consistent from report to report and the number of counties referenced in each successive report is also somewhat variable. These differences, along with the ADDM prevalence in each of the seven reports, are presented in Supplementary File S4. ADDM also computes an overall U.S. ASD prevalence estimate by tabulating all the cases in the participating states from a given report year and dividing by the total 8 year-old population.

### Quantifying ASD Trend Slopes

#### Constant-Age Tracking Versus Age-Resolved Snapshots

The CDDS, IDEA and ADDM reports described above were used to construct the temporal trend in ASD, which was plotted as prevalence versus birth year. Birth year was calculated according to Eq. (1).

1 $$Birth\,Year=Report\,Year-Age,$$

Here we note that Eq. 1 corresponds closely to the Cohort = Period − Age equation used in previous age–period–cohort analyses (Gurney et al. 2003; Keyes et al. 2012). For CDDS and IDEA, which provide a series of annual reports, each of which contains data across a range of ages, the prevalence versus birth year curve can be constructed using two independent approaches: “constant-age tracking” and “age-resolved snapshot” (Nevison 2014). For constant-age tracking, *Report Year* is varied while *Age* is held constant. For the age-resolved snapshot, *Age* is varied while *Report Year* is held constant, using the most recently available report. For ADDM, constant-age tracking is the only approach that can be used, since the ADDM biannual reports provide ASD prevalence estimates only for 8 year-old children.

#### Least Squares Linear Regression and the Null Hypothesis

The slopes of the ASD prevalence versus birth year curves were quantified by least squares linear regression using Matlab R2015b software. With ASD prevalence on the Y-axis and birth year on the X-axis, these slopes reflect the time trend in ASD prevalence, approximated as a linear fit to the data. Hereafter, the terms *b*_*snap*_ and *b*_*track*_ are used to refer to the ASD trend slopes for the age-resolved snapshot and constant-age tracking curves, respectively. The linear regression approach assumes that the ASD prevalence versus birth year relationship can be represented as a linear change over short intervals of data. The errors in the trend slopes were taken from the covariance matrix of the regression.

In analyzing the trend slopes, a key hypothesis tested was the null hypothesis for the age-resolved snapshot slope: is *b*_*snap*_ significantly indistinguishable from 0, i.e., is ASD prevalence independent of birth year? The alternative to the null hypothesis is that *b*_*snap*_ is significantly different from 0, which would indicate a real change in ASD prevalence over time. A *t* statistic was calculated as the ratio of the slope/slope error. Significance was evaluated from a table of critical values of the *t* distribution, with a chosen confidence level of p < 0.01 (Walpole and Myers 1985). The same evaluation was performed for all constant-age tracking slopes *b*_*track*_.

The trend slopes were calculated over selected birth year intervals, as summarized in the results below. These calculations focus on age 8 as the constant age tracked, consistent with ADDM (CDC 2016). The CDDS calculations included the continuous set of 1997–2006 reports plus the 2014, 2016 and 2017 reports, with the latter serving as the most recently available age-resolved snapshot.

For IDEA data, the trend calculations were based on 8 year-old *b*_*track*_ slopes calculated over the 1994–2003 birth year interval. The *b*_*snap*_ slopes were calculated over this same interval from the most recent available IDEA age-resolved snapshot in 2011. Since the IDEA/NCES snapshots have an upper age limit of 17, and we set the lower age limit at the tracking age (i.e., 8), these upper and lower bounds define the 1994–2003 birth year interval of the analysis. The lower age limit is needed to avoid the non-linear rollover that typically occurs at the younger end of an age-resolved snapshot due to underascertainment in very young children (Nevison 2014).

## Results

### California Department of Developmental Services (CDDS) Data Analysis

#### Long-Term Trend Since 1931

**Fig. 1:**
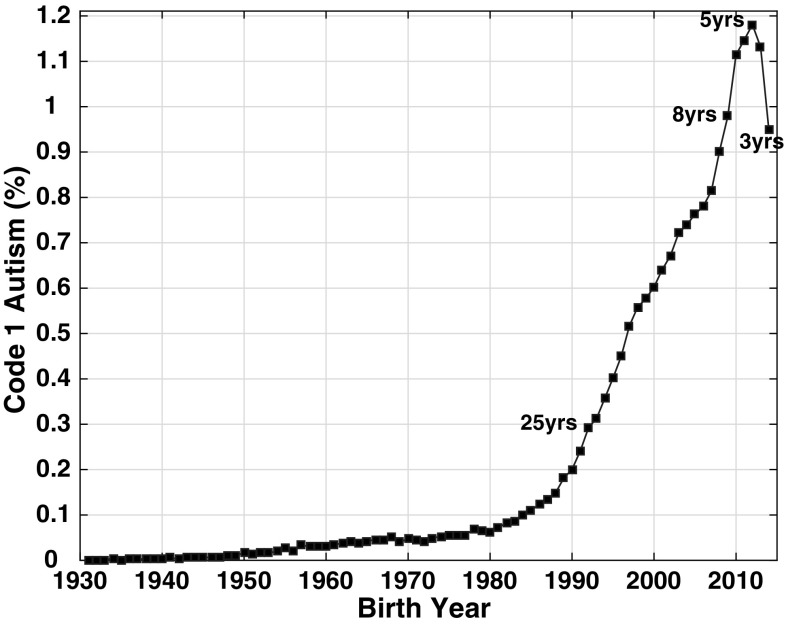
Age-resolved snapshot for 2017, showing the growth in California Department of Developmental Services (CDDS) Code 1 autism prevalence from 0.001% in birth year 1931 to 1.18% in birth year 2012. (Color figure online)

Figure 1 shows an apparent ~ 1000-fold increase in CDDS autism prevalence between birth year 1931, when prevalence was only ~ 0.001%, and birth year 2012, when prevalence had increased to 1.18% among 5 year-olds born in that year. Figure 1 plots the 2017 age-resolved snapshot, which is based on the DSM-5 criteria for ASD with additional CDDS requirements for functional disability in 3 out 7 life challenges.

**Fig. 2:**
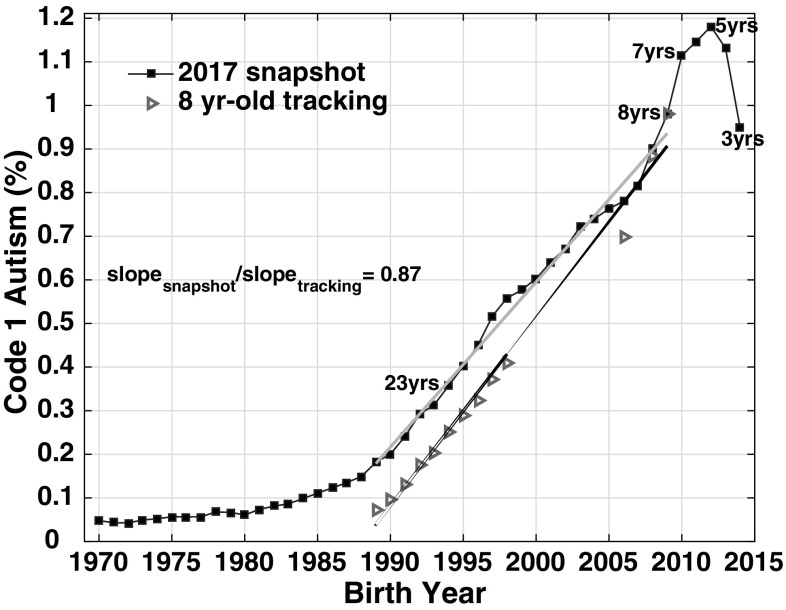
CDDS data from 1997 to 2006, 2014, 2016 and 2017 reports, comparing 8 year-old tracking (red triangles) to 2017 age-resolved snapshot (blue squares) slopes over birth year interval 1989–2009. The *b*_*snap*_:*b*_*track*_ slope ratio, representing the ratio of the grey:black slopes, is 0.87. Selected ages are labeled on the blue age-resolved snapshot curve, indicating the age of each birth cohort in 2017. (Color figure online)

#### Snapshot and Tracking Slopes

The snapshot versus tracking slope analysis is focused on the most recent decades of the CDDS data from 1989-present. This period covers school-age children, who are more likely than adults to be re-evaluated periodically for ASD, given the Child Find legal mandate (Wright and Wright 2007) and parental motivation of publicly-funded services. The availability of successive annual reports in these recent decades also allows for intercomparison of the age-resolved snapshot and constant-age tracking methods for estimating time trends. These recent time trends can be approximated with linear fits and thus used to quantify trend slopes. For the birth year 1989–2009 interval, the 2017 *b*_*snap*_ trend slope (3.8 ± 0.08 per 10,000 per year) is significantly greater than 0 at a high confidence level (p ≪ 0.01), suggesting that the data are inconsistent with the null hypothesis that Code 1 autism is a constant prevalence condition (Fig. 2). The constant-age tracking slope *b*_*track*_ (4.3 ± 0.15 per 10,000 per year) also differs significantly from 0 at a high confidence level (p ≪ 0.01).

The CDDS *b*_*snap*_ slope is somewhat flatter than the *b*_*track*_ slope computed over the same 1989–2009 birth year interval. The flatter slope is the result of upward revision over time in Code 1 autism prevalence for the earlier birth cohorts in the snapshot. For example, in the 1997 CDDS report, prevalence among 8 year-olds born in 1989 was 0.08%, but had been revised upward to 0.18% in the 2017 CDDS report for this same 1989 birth cohort. Regardless of which of these starting points is used, the growth to a prevalence of 0.98% among 8 year-olds in the 2009 birth cohort represents a substantial increase. The *b*_*snap*_:*b*_*track*_ slope ratio over the 1989–2009 birth year interval is 0.87, suggesting the trend slopes are largely consistent across the constant-age tracking and age-resolved snapshot methods (Fig. 2).

### Evolution of the CDDS Snapshot

**Fig. 3:**
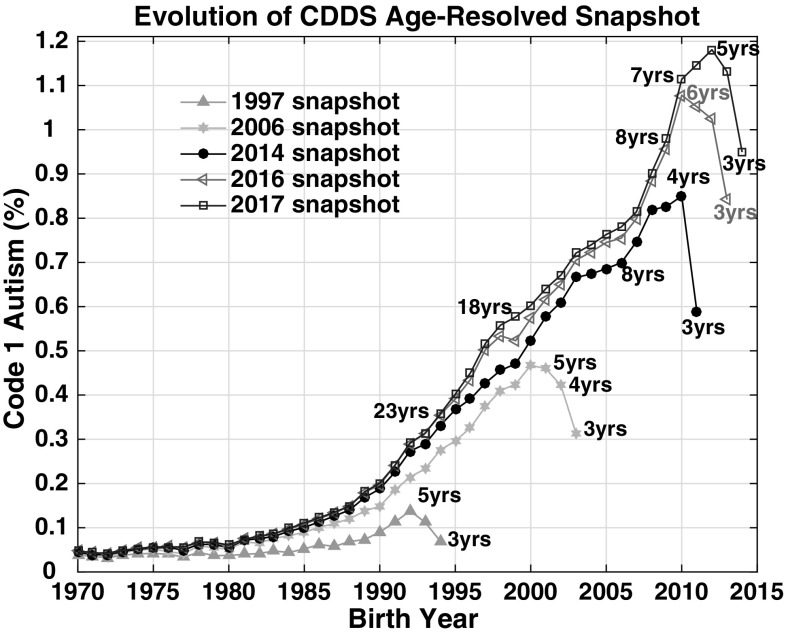
CDDS Code 1 autism data comparing 1997 (green triangles), 2006 (cyan squares), 2014 (black circles), 2016 (magenta triangles) and 2017 (blue squares) age-resolved snapshots. Selected ages are labeled on each snapshot curve, indicating the age of each birth cohort at the time each respective CDDS report was compiled. (Color figure online)

Code 1 autism prevalence in the CDDS age-resolved snapshots has evolved substantially over time from the earliest report in 1997 to the most recent report in 2017 (Fig. 3). The 1997 age-resolved snapshot is relatively flat through the 1970s but prevalence starts trending upward around the mid to late 1980s, reaching a high of 0.14% among 5 year-olds born in 1992. Moving ahead 9 years, prevalence has increased overall in the 2006 snapshot from about birth year 1975 onward and reaches a high of 0.47% among 6 year-olds born in 2000. Another 8 years later, prevalence has increased again in the 2014 snapshot, with proportionally more growth for the more recent (after 1985) birth cohorts than the older birth cohorts, and reaches a high of 0.86% among 4 year-olds born in 2010. Additional increases occur in the 2016 snapshot, mainly after birth year 1995, with peak prevalence (1.08%) still occurring among the 2010 birth cohort, now age 6. One year later in the 2017 snapshot, peak prevalence (1.18%) shifts to 5 year-olds born in 2012.

Visual inspection of the complete 2017 snapshot (Fig. 1) suggests that Code 1 autism prevalence has been creeping slowly upward from near-zero levels since at least 1940, reaching 0.06% in 1980, at which point the numbers started rising more quickly, tripling to 0.18% by 1989. The change point at which the even more rapid increase of the 1990s and 2000s began is debatable, but appears to have occurred between 1988 and 1990. The 2014, 2016 and 2017 snapshots all suggest a slower rate of growth in the late 1990s and mid 2000s (Figs. 1, 3). However, all three snapshots show that the rate of growth accelerated again after 2006 until prevalence had reached an all-time high of 1.18% of 5 year-old children born in 2012.

### Cohort Analysis

**Fig. 4:**
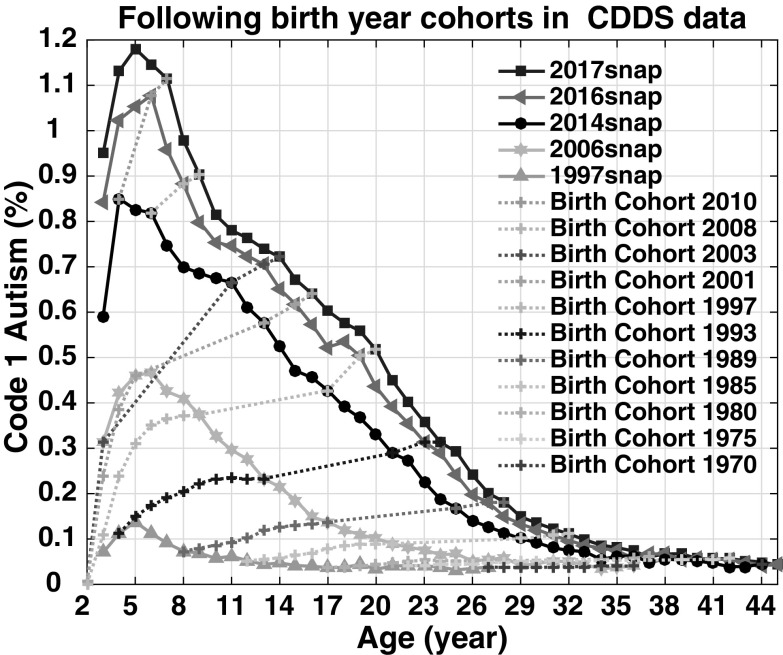
CDDS prevalence by age, period, and year of birth. Dotted lines represent children born in the denoted year as they age over time. The period curves use the same data as the age-resolved snapshots for 1997, 2006, 2014, 2016 and 2017 in Fig. 3, but are plotted versus age (i.e., in reverse order along the X-axis) rather than versus birth year. (Color figure online)

A conventional cohort–period–age plot (Gurney et al. 2003) provides an alternative, complementary way to examine how prevalence among selected birth cohorts has evolved as they age. Figure 4, which follows 11 CDDS birth cohorts over time, shows an upward revision with age starting among the birth cohorts of the late 1980s. Prevalence increases rapidly between ages 2 and 8. After age 8, a flatter but still ongoing upward revision continues throughout the teenage years and into adulthood. For example, prevalence in the 1997 birth cohort rises by 0.15% from age 8 to 20, while prevalence in the 1989 birth cohort rises by 0.11% from age 8 to 28. However, the cohort–period–age plot suggests little upward revision with age among the birth cohorts of the 1970s and early 1980s, consistent with Fig. 3.

### Comparison of CDDS, IDEA and ADDM 8 Year-Old Prevalence in 16 States

**Fig. 5:**
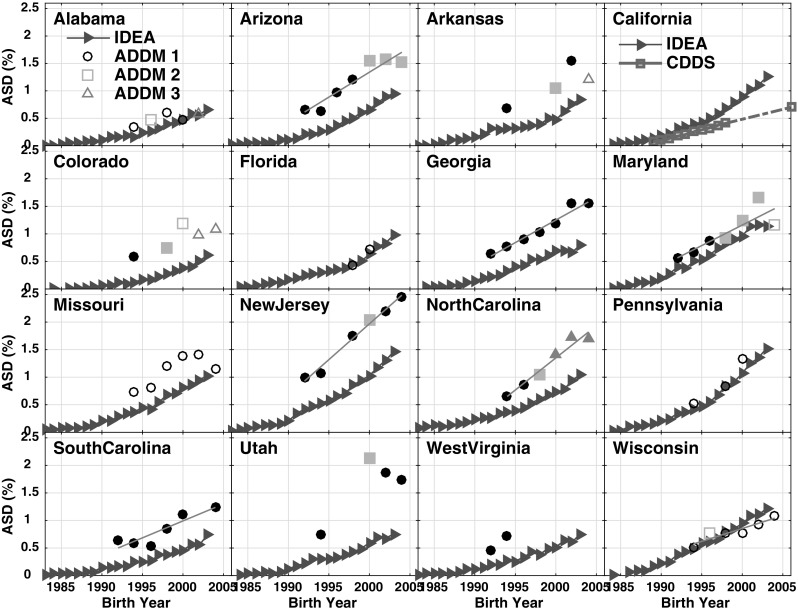
Comparison of data tracking ASD prevalence among 8 year-olds from 3 different networks. IDEA data (red triangles) tracking 8 year-olds over report years 1991–2011 (corresponding to birth years 1983–2003) are available for all states. In California, the IDEA data are compared to CDDS 8 year-olds (magenta squares) tracked from report years 1997–2006 and 2014 (birth years 1989–1998 and 2006). For all other states, the IDEA data are compared to ADDM 8 year-olds tracked biannually from birth years 1992–2004. Up to 3 different black or grey symbols are used for the ADDM data to denote shifts and inconsistencies in the number of counties sampled within each state in successive reports. In addition, the ADDM data are plotted as solid symbols for prevalence derived based on both health and education records and as open symbols when only health records were available. (Color figure online)

Expanding the analysis to the IDEA and ADDM datasets allows examination of ASD trends in states beyond California and among different networks that often include a larger share of milder ASD than CDDS. Here, in order to compare all 3 networks, we show 8 year-old tracking trends, rather than age-resolved snapshot trends, because only the former are available for ADDM. Figure 5 and Table 2 show that ASD prevalence varies among states, and also between different datasets within a given state. For IDEA, ASD prevalence among 8 year-olds born in 2003 (the most recent birth cohort available) varies over more than a factor of 2 among the 16 states examined, ranging from 0.61% in Colorado to 1.53% in Pennsylvania. For ADDM, ASD prevalence among 8 year-olds in the most recently available 2004 birth cohort also varies by more than a factor of 2, ranging from 1.08% in Wisconsin to 2.46% in New Jersey. Moreover, the New Jersey prevalence is more than 4 times larger than the Alabama prevalence of 0.57% (reported most recently for the 2002 birth cohort) (CDC 2014). In California, both absolute prevalence and the rate of increase in prevalence are greater in IDEA than in CDDS. In the remaining states, ADDM prevalence exceeds IDEA prevalence in 11 of 15 overlapping states and is comparable in 4 states.

**Table 2:** ASD prevalence slopes: ADDM and IDEA snapshot versus tracking

State	Trend slope ± slope error (per 10,000 per year)^a^			IDEA slope ratio^e^ *b*_*snap*_/*b*_*track*_	Recent 8 year-old prevalence (%)	
	ADDM^b,c^ *b*_*track*_	IDEA^d^ *b*_*snap*_	IDEA^d^ *b*_*track*_		ADDM (BY 2002 or 2004)^f^	IDEA (BY 2003)
Alabama		4.0 ± 0.9	5.4 ± 0.2	0.74	0.57	0.66
Arizona	9.1 ± 1.5	5.9 ± 0.3	8.0 ± 0.3	**0.74**	1.52	0.96
Arkansas		4.1 ± 0.6	5.8 ± 0.8	0.71	1.20	0.84
California	N/A	7.6 ± 0.2	9.7 ± 0.4	**0.78**	N/A	1.25
Colorado		3.4 ± 0.3	5.2 ± 0.4	**0.67**	1.08	0.61
Florida		6.6 ± 0.5	7.6 ± 0.7	**0.87**	N/A	0.98
Georgia	8.2 ± 0.8	3.4 ± 0.8	4.8 ± 0.4	0.70	1.55	0.80
Maryland	7.4 ± 2.0	5.0 ± 0.9	8.2 ± 0.5	0.61	1.16	1.15
Missouri		5.0 ± 0.6	7.5 ± 0.4	0.67	1.15	1.03
New Jersey	13 ± 0.9	9.8 ± 0.6	10.4 ± 0.6	**0.94**	2.46	1.46
No. Carolina	12 ± 1.3	5.0 ± 0.4	7.5 ± 0.4	**0.67**	1.69	1.04
Pennsylvania		9.6 ± 0.5	12.6 ± 0.5	**0.76**	N/A	1.53
S. Carolina	6.1 ± 1.4	4.0 ± 0.3	5.1 ± 0.5	**0.78**	1.24	0.74
Utah		2.0 ± 0.3	5.6 ± 0.4	0.36	1.73	0.76
W. Virginia		4.3 ± 0.7	5.2 ± 0.4	0.82	N/A	0.76
Wisconsin	4.7 ± 0.9	6.0 ± 0.5	8.1 ± 0.3	**0.74**	1.08	1.22

^a^To convert to %/year, divide by 100

^b^The ADDM 8 year-old tracking slope is reported only when the least squares linear regression slope *b*_*track*_ is statistically different from 0 at a confidence level of p < 0.01 or better

^c^For ADDM data, the birth year span ranges from as early as 1992 to as late as 2004. See Fig. 5 and Supplementary File S4 for individual state details

^d^For all IDEA data, the birth year span is 1994–2003, the tracking age is 8 years old and the 2011 IDEA snapshot age range is 8–17 years old

^e^ *b*_*snap*_/*b*_*track*_ slope ratios are shown in bold face, indicating smaller uncertainty, when the slope error is ≤ 10% of the regression slope for both b_track_ and b_snap_

^f^Birth Year (BY) 2004 prevalence shown if available, otherwise BY 2002

All 2011 IDEA *b*_*snap*_ trend slopes presented in Table 2 are significantly > 0, suggesting the IDEA data are inconsistent with the null hypothesis that ASD is a constant prevalence condition over time. The IDEA *b*_*snap*_ slope errors are considerably larger than for the CDDS data (ranging from 3 to 25% of *b*_*snap*_ with a median of 9%), but the *t* statistic still indicates that IDEA *b*_*snap*_ is non-zero at the p < 0.01 confidence level for all 16 states in Table 2. Similarly, all IDEA 8 year-old tracking slopes *b*_*track*_ are significantly > 0 (p < 0.01) in all 16 states, with slope errors ranging from 4 to 14% (median 6%) of *b*_*track*_. Interestingly, however, the ADDM *b*_*track*_ slopes differ significantly from 0 in only 7 out of the 15 ADDM states in Table 2.

**Fig. 6:**
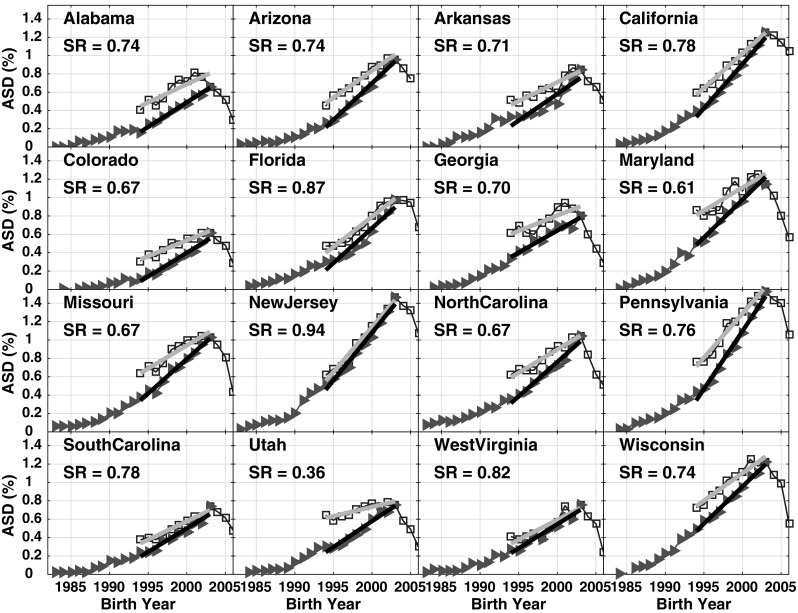
IDEA data from 1991 to 2011 reports, comparing 8 year-old tracking data (red triangles) to 2011 IDEA age-resolved snapshot data (blue squares). The slopes of the ASD prevalence increase over birth year interval 1994–2003 are determined by least squares linear regression and plotted as gray and black lines for the 2011 snapshot and 8 year-old tracking, respectively. The *b*_*snap*_:*b*_*track*_ slope ratio (*SR*) is shown in each panel, representing the ratio of the grey:black slopes. (Color figure online)

The IDEA *b*_*snap*_ trend slopes are invariably flatter than the *b*_*track*_ slopes in all 16 states, with *b*_*snap*_:*b*_*track*_ slope ratios ranging from 0.36 in Utah to 0.94 in New Jersey (Fig. 6). Excluding Utah, which is an outlier compared to the other states, the mean *b*_*snap*_:*b*_*track*_ slope ratio is 0.75 ± 0.085. In California, the ratio is 0.78.

### Nationwide ASD Prevalence Trends Among 8 Year-Olds

**Fig. 7:**
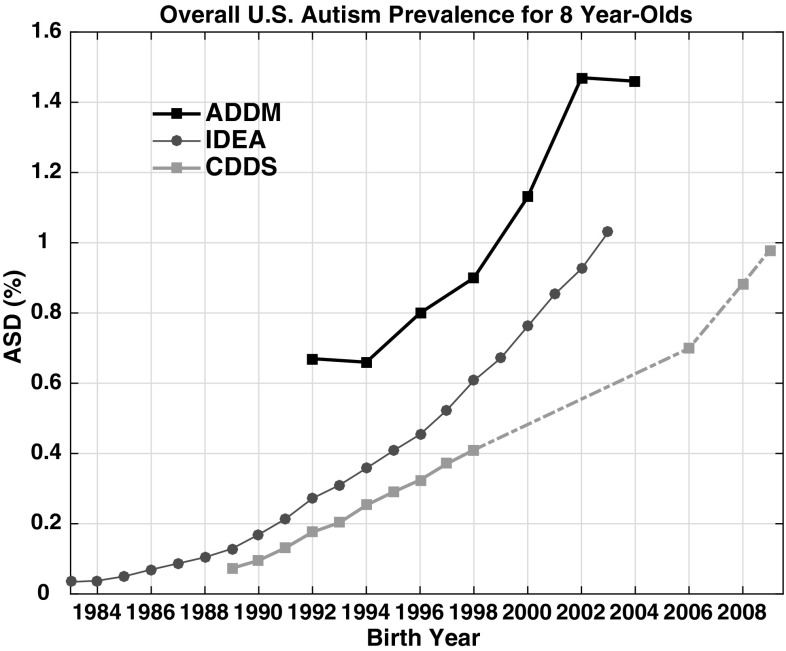
Comparison of data tracking overall U.S. ASD prevalence among 8 year-olds from the IDEA and ADDM networks. Also shown are the CDDS 8 year-old data (covering CDER Code 1 autism cases in California only). (Color figure online)

The 8 year-old tracking curves for United States nationwide ASD prevalence differ across networks, just as they did among the 16 individual states (Fig. 7). Nationwide ADDM prevalence is a factor of 1.5–2.5 higher than IDEA over the overlapping 1992–2003 birth year interval. The two datasets follow a similar trend slope over most of this interval, with notable exceptions between 1992–1994 and 2002–2004. ADDM suggests a flat trend over these periods, while IDEA 8 year-old prevalence climbs from 0.27 to 0.36% for 1992–1994 and from 0.92% in 2002 to 1.03% in 2003. CDDS data, which are plotted in Fig. 7 for comparison although they are for California only, also increase from 1992 to 1994, with a trend slope similar to that of IDEA. Although there is a data gap in the CDDS 8 year-old tracking data from birth year 1998–2006, the 2014, 2016 and 2017 CDDS reports suggest that prevalence continues to increase over those gap years.

## Discussion

### California Department of Developmental Services (CDDS)

#### Overview of Trends and Current Prevalence

The CDDS data featured in Figs. 1, 2, 3, 4 are widely considered the most reliable long-term record of autism prevalence trends in the United States (McDonald and Paul 2010; Autism Society San Francisco Bay Area 2015). The prevalence versus birth year curves suggest a dramatic increase over time, especially when viewed in the context of the full span of data extending back to birth year 1931, when the reported prevalence was only ~ 0.001% (Fig. 1). While a substantial fraction of the 1931 birth cohort was likely deceased by the time of the 2017 snapshot, this cohort had the same low prevalence at the time of the 1997 snapshot, when it was only in its 60s (Supplementary File S1). The increase from ~ 0.001 to 1.18% in the 2012 birth cohort has occurred gradually, with a slow upward creep starting as far back as the 1940s, but with several change points along the way, around ~ 1980, ~ 1990, and ~ 2007, when the rate of growth accelerated.

The interpretation of the data is complicated by the redefinition of CDDS Code 1 from “autism, full syndrome” to AD in 2008, and by the further redefinition from AD to ASD at the end of 2014. The ~ 1980 and ~ 1990 change points noted above are relatively insensitive to these code changes, while the most recent birth cohorts are more likely to be affected. Although the entire 2017 snapshot in principle has been updated to the new DSM-5 definition of Code 1, due to the annual to triannual diagnosis update schedule, the point of entry into the CCDS system may be the single most important time for diagnosis; the CDDS regional centers in some cases may renew the date stamp of the Client Development Evaluation Report (CDER) without actively reviewing the diagnosis (Paul Choate, personal comm.). It is therefore possible that some of the new cases entering under Code 1 in the 2016 and 2017 snapshots would have been diagnosed with PDD-NOS or Asperger’s under the earlier DSM-IV criteria and thus would not have been allowed into the CDDS caseload.

However, several considerations argue against changing diagnostic criteria as the only or primary cause of the new uptick in prevalence in recent years, which started around birth year 2007. First, the 2007 uptick is evident already in the 2014 snapshot, in which Code 1 was defined as AD based on DSM-IV. Second, the CDDS caseload historically has covered the more severe end of the ASD spectrum and likely continues to do so. The CDDS screening process is stringent in that it requires not only an autism diagnosis to qualify for services, but also demonstration of “significant functional disability” in at least 3 out of 7 life challenge areas. CDDS in fact raised the bar on this requirement in 2003 from a previously more lenient standard of 1 out of 7 (Autism Society San Francisco Bay Area 2015). This increased stringency makes the upward surge in prevalence around birth year 2007 even more remarkable. Third, although some milder cases may be allowed in initially as very young children, the annual to triannual diagnosis update process screens them out relatively quickly. Of 34,711 new Code 1 cases entering CDDS services after November 2014, nearly 3000 have subsequently been discharged, due either to moving out of state or no longer meeting the qualifying requirements (Paul Choate, personal comm.). The latter is the more likely reason, since CDDS historically has had a substantially lower attrition rate. Of those new cases still qualifying for services, 68% had an ASD impact scale rating of moderate, 7.5% severe and 24% mild.

#### Snapshot Evolution and Snapshot Versus Tracking Analysis

The thirteen different age-resolved reports spanning 1997–2017 provide a means to examine how the diagnosed prevalence of CDDS autism has evolved over the years among the same birth cohorts. The passage of time across this 21-year span of reports has allowed extensive opportunities for retroactive diagnosis, during an era of growing awareness, improved educational services and increasing availability of therapies and treatments. Accordingly, the birth cohorts from the late 1980s onward are characterized by modest but ongoing increases in diagnosed autism prevalence. These increases are evident in the evolution of the CDDS reports over time (Fig. 3), as well as the growth in prevalence among individual birth cohorts with age (Fig. 4).

Autism by definition is either present from birth or has been expressed by age 3. However, even using as a baseline the prevalence at age 8 [the tracking age used by ADDM (CDC 2016)], Fig. 4 shows an ongoing increase in prevalence into adulthood by factors ranging from 1.35 to 2.5 (corresponding to absolute rises in prevalence of 0.11–0.15%). Part of this increase may be due to net migration into California (Fountain and Bearman 2011; Paragon Real Estate Group 2017), which is not accounted for in our prevalence calculation, since we use static live birth data for the denominator. Still, the ongoing upward revision is curious and lends support to claims that better and expanded diagnosis is driving at least part of the reported increase in these later birth cohorts (assuming the increase is not explained by immigration). However, the increases of up to a factor of 2.5 over time from age 8 onward among individual birth cohorts (Fig. 4) are small compared to the much larger increases that have occurred with time across successive birth cohorts. Across birth cohorts in the 2017 snapshot, CDDS autism prevalence has increased by a factor of 25 from birth years 1970–2012 and by a factor of 1000 from birth years 1931–2012.

Focusing on the most recent birth years of 1989–2009, the age-resolved snapshot versus 8 year-old tracking analysis shows that the trend slopes computed from these two independent methods are largely consistent. Both methods indicate a steep increase over time in Code 1 autism prevalence, with a slope ratio of 0.87 (Fig. 2). Nevison (2014) suggested that the *b*_*snap*_:*b*_*track*_ slope ratio of an autism prevalence versus time graph provides a rough estimate of the real fraction of the constant-age tracking trend. That interpretation, based on the slope ratio of 0.87, would suggest that 87% of the increase in CDDS autism tracked among 8 year-olds over the 1989–2009 birth year interval is due to a true rise in the condition. A caveat here is that the *b*_*snap*_:*b*_*track*_ ratio is sensitive to tracking age. When we repeat the calculations in Fig. 2, but with 6 or 7 instead of 8 as the tracking age, we calculate *b*_*snap*_:*b*_*track*_ ratios of 82 and 86%, respectively, over birth years 1991–2011 and 1990–2010. One reason for the sensitivity of the *b*_*snap*_:*b*_*track*_ ratios to tracking age may be that the assumption of linearity, which is required for the calculation, starts to break down in recent years due to the new uptick in the prevalence data around birth year 2007. While the *b*_*snap*_:*b*_*track*_ method involves substantial uncertainty, it nevertheless provides an empirical, quantitative estimate of the real fraction of the increase in autism across CDDS birth cohorts over time, suggesting that ~ 82–87% of the tracked increase since birth year ~ 1990 may be due to a true rise in the condition. By implication, the residual ~ 13–18% of the increase is likely not real and may be due instead to immigration or better and expanded diagnosis.

There are three main hypotheses for how better and expanded diagnosis might lead to an apparent but non-real increase in autism prevalence (Keyes et al. 2012): (1) diagnostic substitution, with intellectual disability typically named as the diagnostic substituent (Croen et al. 2002; Polyak et al. 2015), (2) diagnostic expansion, which often refers to the addition of Asperger’s syndrome to the list of autism spectrum disorders, and (3) diagnostic oversight, which refers to the possibility that children who were overlooked in years past are now being identified, thanks to increased awareness among families and diagnosticians (Blaxill 2004).

Hypothesis 1 is unlikely, since the trend in intellectual disability in California has been more or less flat over the time frame of the steep rise in autism (Shattuck 2006; Nevison and Blaxill 2017). Hypothesis 2 may be a contributing factor, given the several revisions to CDER Code 1 over the years, but is unlikely to be the main driver of the increases shown in Figs. 1, 2, 3, for the reasons discussed earlier. The third hypothesis, diagnostic oversight, also may be a viable explanation for some of the upward revision over time among specific birth cohorts shown in Figs. 3 and 4. However, we cannot ascribe the upward revision definitively to any specific hypothesis based on the high-level statewide data presented in this study.

In contrast to the birth cohorts from the late 1980s onward, there is little or no upward revision in diagnosis among the birth cohorts of the 1930s, 1940s and 1950s, and only small upward revision for the birth cohorts of the 1960s, 1970s and early 1980s (Figs. 3, 4, Supplementary File S1). The CDDS datasets thus show no obvious evidence of a large, overlooked population of autistic adults in the 1931 through early 1980s birth cohorts. However, these cohorts were already in their teens or older by the time of the first available CDDS report in 1997. Thus, these cohorts, with the exception of the teenagers born in the early 1980s, in general were not covered by the Child Find mandate and it is unclear whether they would have the opportunity or incentive to be evaluated for ASD as adults if they were not already diagnosed as children.

### Differences Among States and Data Networks

Previous studies based on assemblages of autism data from different places, reflecting different criteria, and grouped into irregular time or age bins have been unable to confirm a clear trend in the data. Further, the greater than threefold difference among 8 year-olds in an (early) ADDM survey between New Jersey and Alabama has been cited as a reason why changing diagnostic criteria have played a major role in creating the apparent increase in autism (Fombonne 2009). Our study concurs that autism prevalence estimates differ substantially among CDDS, IDEA and ADDM datasets and among states within the IDEA and ADDM networks. These differences, combined with the likelihood of strong gradients in prevalence as a function of age, suggest the need for caution in combining widely disparate datasets when evaluating time trends in autism prevalence or, more generally, when citing a single number [e.g., 1 in 68 (CDC 2016)] as the overall rate of autism.

A comparison of New Jersey and Alabama is instructive for understanding some of the reasons behind the large apparent differences in ASD prevalence among states and data networks. Our analysis also finds a more than threefold difference between ADDM prevalence in New Jersey versus Alabama in the most recent available common birth year, 2002 (Fig. 5), but much of the difference can be attributed to several identifiable factors. First, 8 year-olds are still substantially under-ascertained in Alabama while in New Jersey they are not. For the 2001 birth year cohort, comparison of 8 year-old and 10 year-old IDEA data (Supplementary Dataset S3) shows that prevalence among Alabama children was revised upward by 40% from age 8 to 10, whereas in New Jersey 8 year-olds were more fully diagnosed, with only 4% upward revision from age 8 to 10.

A second factor involves differences in the regions sampled within each state. Taking birth year 2002 as an example, prevalence among 8 year-olds differs by a factor of 3.8 for ADDM between New Jersey and Alabama, but IDEA prevalence among 8 year-olds differs by only a factor of 2.3 between the two states. IDEA data cover the whole of both states. In contrast, ADDM typically surveys 4 urban counties in New Jersey, but samples Alabama broadly across 32 counties in the northern half of the state, comprising a mix of urban and rural areas. ASD prevalence in general tends to be higher in urban than rural areas, for reasons that are not clear but may involve enhanced exposure to beneficial environmental microbes in rural areas and/or higher levels of toxins in urban areas (Becker 2010; Dickerson et al. 2016). As a result, the different sampling strategies across the two states will tend to exaggerate the difference in ASD prevalence between New Jersey and Alabama.

If one combines these two factors (i.e., comparing at age 10 instead of age 8, and comparing IDEA prevalence across the whole of both states instead of ADDM prevalence in selected counties), one can account for much of the difference in ASD prevalence between New Jersey and Alabama. Indeed, the prevalence ratio is reduced from 3.8 to 1.5. This remaining ratio of 1.5 may or may not reflect a true regional difference between the two states. Regardless, each state individually shows a statistically significant increasing trend in ASD, and that trend furthermore is relatively consistent between the age-resolved snapshot and constant age tracking methods, when applied to IDEA data (Fig. 6; Table 2).

Another important consideration is that, within the ADDM network, New Jersey has more detailed and extensive records available and has access to information from both education and health sources in its ascertainment, whereas Alabama’s case finding is limited to healthcare sources (CDC 2014). New Jersey’s unusual alignment of snapshot and constant-age tracking slopes (cf. *b*_*snap*_:*b*_*track*_ ratio of 0.94 shown in Fig. 6) may indicate that New Jersey’s educational records are more comprehensive than those in other states, leading to more complete case finding. Here it is interesting and somewhat paradoxical to note that the IDEA and ADDM prevalence values tend to agree best in states in which the ADDM prevalence is estimated only based on health records (Alabama, Missouri, Wisconsin and Pennsylvania) (CDC 2014), while ADDM prevalence tends to exceed IDEA prevalence in states where ADDM has access to both health and educational records (Fig. 5). A detailed analysis in Utah found that ASD prevalence was higher when estimated based on both health and education data, with a greater proportion of cases ascertained from health records (Pinborough-Zimmerman et al. 2012). That analysis may explain part of the large discrepancy between ADDM and IDEA prevalence in Utah shown in Fig. 5.

Another important factor in these considerations is the extent to which milder forms of ASD are included in the definition of autism. Among the three data networks examined in this paper, autism prevalence increases in the following order: CDDS < IDEA ≤ ADDM (Figs. 5, 7). This ordering is broadly consistent with the inclusion or exclusion of milder ASD in these datasets. CDDS historically has been entirely AD and continues to focus on the more severely affected population under *DSM-5*, IDEA includes some milder forms of ASD in some states but not in others (MacFarlane and Kanaya 2009), while ADDM attempts to include all ASD subtypes. Here, it is notable that Asperger’s cases have accounted consistently for only ~ 10% of the total ADDM cases over the reports (birth year 2000–2004) that provide this information (CDC 2012, 2014, 2016). However, given that the median age of Asperger’s diagnosis is about 8 (Lingam et al. 2003), ADDM, which surveys 8 year-olds, likely misses the true number of Asperger’s cases.

### Inconsistencies in the ADDM Network and in Nationwide Prevalence

While ADDM data are commonly cited as the definitive metric of United States ASD prevalence, only 7 out of 15 ADDM states, as shown in Fig. 5, have an 8 year-old tracking slope *b*_*track*_ that is statistically different from 0 at the p < 0.01 confidence level. This was surprising because we had expected to be able to reject the null hypothesis for all *b*_*track*_ slopes simply due to better and expanded ASD diagnosis over time. However, inconsistencies in the ADDM Network, as well as the small number of data points and missing years of data, may have contributed to the surprising ADDM *b*_*track*_ results. Among the 15 states surveyed over the history of the ADDM network, only 1 (Georgia) has consistently monitored ASD in the same subset of counties with complete coverage, including access to health and education data, over all seven available ADDM reports from birth year 1992–2004.

Another curious feature of ADDM data is that the network appears to underestimate the upward trend in nationwide ASD prevalence between birth years 1992–1994 and again between birth years 2002–2004 compared to IDEA and CDDS (Fig. 7). The ADDM numbers remain stable over both of these 2-year intervals, even as CDDS and IDEA prevalence continues to increase. One reason for the flat ADDM trend from 2002 to 2004 may have been that 2 states (Maryland and Arkansas), which had traditionally had access to health and education records in earlier ADDM reports, lost access to most of their education records in 2004 (CDC 2014, 2016). Accordingly, both these states reported a decrease in ASD prevalence from 2002 to 2004.

The flat ADDM trend from 1992 to 1994 trend may reflect the sensitivity of the nationwide ADDM mean to the changing set of counties and states surveyed. The overall mean is computed as the sum of all the ASD cases in the participating states divided by the sum of the total population surveyed. In both 1992 and 1994 the ADDM overall mean was about 0.66%, or 1/150. In 1992, six states were sampled, while in 1994, 14 states were sampled, including all original 6. If just the original 6 states had been sampled for birth year 1994, instead of remaining flat, the overall mean would have increased by 10% from 1992 to 0.74%. Each of the subsequent ADDM reports has brought a new shift in which and how many states are included. Each also has been accompanied by changes in the number of counties sampled within many of the participating states (Supplementary File S4). Given these uncertainties, it is unfortunate that restrictions on the availability of cohort-referenced IDEA data beyond the 2011 report will hinder systematic tracking of IDEA trends into more recent birth years, making it difficult to compare future IDEA and ADDM nationwide trends.

## Conclusion

CDDS autism prevalence has risen dramatically over the last 35 years, increasing from ~ 0.05% in birth year 1970 to nearly 1.2% in birth year 2012. The available data extending back to 1931 show a prevalence of only 0.001% in that birth cohort. Prevalence slowly increased from ~ 1940 to 1980, at which time the first of several change points occurred, in ~ 1980, ~1990, and ~ 2007, each associated with a new uptick in the rate of growth. The CDDS dataset suggests that prevalence has increased by a factor of 25 from birth year 1970–2012 and by as much as a factor of 1000 from birth year 1931–2012.

CDDS continues to exclude most milder cases of autism, despite two different changes to its diagnostic criteria in the last decade. As a result, IDEA autism prevalence in California is substantially higher than CDDS prevalence. ADDM ASD prevalence in turn is substantially higher than IDEA prevalence in 11 out of 15 overlapping states, likely due to a combination of factors, including inclusion of all forms of ASD, access to health and education-based records, and disproportionate sampling of urban over rural areas in some states. While about half of ADDM states have non-significant 8 year-old tracking trend slopes, this is attributable in part to discontinuous or inconsistent data records and differences in completeness, suggesting the need for more consistent sampling strategies when evaluating time trends in overall ASD prevalence. The ADDM network states with the most consistent access to information from multiple (health and education) sources show the most strongly increasing ASD trends. Metropolitan New Jersey, for example, has been the leading indicator of autism prevalence in the ADDM network across the decade, with the most recent prevalence estimate showing ASD prevalence as high as 2.5% among 8 year-olds of the 2004 birth cohort (Zahorodny et al. 2014; CDC 2016).

## Electronic supplementary material

Below is the link to the electronic supplementary material.

Supplementary File S1 presents California autism counts and prevalence data from CDDS for report years 1997-2006, 2014, 2016 and 2017 (XLS 72 KB)

Supplementary File S2 presents IDEA autism counts for all 50 states, tracked among 5 through 17 year-olds for report years 1991-2011 (XLSX 147 KB)

Supplementary File S3 is the same as Supplementary File S2, except the IDEA data are in units of prevalence, i.e., autism count per 10,000 (XLSX 144 KB)

Supplementary File S4 presents biannual estimates of autism prevalence among 8 year-old for birth years 1992-2004 in 15 states from the ADDM Network (XLSX 45 KB)

Supplementary material 5 (DOCX 26 KB)
