# Supplementary material for: California Autism Prevalence Trends from 1931 to 2014 and Comparison to National ASD Data from IDEA and ADDM
Source: J Autism Dev Disord. 2018 Jul 5;48(12):4103–17. doi: 10.1007/s10803-018-3670-2 (PMC6223814; doi:10.1007/s10803-018-3670-2)
Supplement: Supplementary file 5 — Supplementary material 5 (DOCX 26 KB) [file 10803_2018_3670_MOESM5_ESM.docx]

**Supplementary Information**

**California autism prevalence trends from 1931-2014 and comparison to national ASD data from IDEA and ADDM**

**Cynthia D. Nevison**

Institute for Arctic and Alpine Research, Campus Box 450, University of Colorado, Boulder, Colorado 80309-0450, [Cynthia.Nevison@colorado.edu](mailto:Cynthia.Nevison@colorado.edu), Tel. 303 492-7924.

**Mark Blaxill**

Health Choice, Cambridge, Massachusetts, mblaxill@comcast.net

**Walter Zahorodny**

Rutgers New Jersey Medical School, Newark, New Jersey, zahorodn@njms.rutgers.edu **Table of Contents**

S1. CDDS Code 1 autism prevalence for California 2

S2. IDEA autism counts for the 50 United States + D.C. 2

S3. IDEA/NCES autism prevalence for 50 states + D.C. 3

S4. ADDM autism prevalence in 15 states 3

**S1 Dataset. CDDS autism counts and prevalence for California**

Supplementary File S1 presents Code 1 autism counts and prevalence data from the California Department of Developmental Services. Autism prevalence is computed by dividing the autism counts by California live births. The live birth data are included in the file. Prevalence is presented in units of cases per 10,000. (To convert to percent, divide by 100.) The data are organized in a series of columns, each representing an age-resolved snapshot for a given report year, from 1997-2006, 2014, 2016 and 2017. Each row corresponds to birth year and the columns are shifted for each report year such that each row corresponds to the same birth year. The age of the birth cohort at the time of the 2014, 2016 and 2017 reports are given in the final columns, and can be readily calculated for any of the earlier snapshots as: *Age = Report Year - Birth Year*.

The presentation of the CDDS data lends itself most easily to the calculation of age-resolved snapshot trends. However, constant-age tracking trends can be computed by reading in the data across an upward diagonal from 1997-2006 for a selected age and adding the appropriate additional data points for 2014, 2016 and 2017.

**S2 Dataset. Statewide IDEA autism counts**

Supplementary File S2 presents autism counts from the Individuals with Disabilities Education Act (IDEA) database, broken down by state and tracked among 5 through 17 year-olds from report years 1991-2011. These data were formerly available at ([www.ideadata.org](http://www.ideadata.org)), but are no longer easily accessible following recent changes to the IDEA website, in which data are no longer made available in fully age-resolved format. The data in S2 were downloaded and archived by the authors prior to these changes.

The data are presented as a series of 13 Excel spreadsheets, with 51 rows and 21 columns. The rows correspond to each of the 50 United States plus the District of Columbia, arranged in alphabetical order. The columns correspond to the 21 report years, arranged from 1991 to 2011. A separate spreadsheet is included for each age from 5 to 17. The arrangement of the spreadsheets is organized to allow constant-age tracking trends to be read in directly across a row for a given state. The presentation of the IDEA differs from that of the CDDS data in S1, which are in columns corresponding to age-resolved snapshots. However, age-resolved snapshot trends can be computed readily from the IDEA data by reading in all 13 sheets and selecting a report year of interest.

**S3 Dataset. Statewide IDEA autism prevalence**

Supplementary File S3 is presented in the same format as Supplementary File S2, except the data are in units of prevalence, i.e., autism count per 10,000. (To convert to prevalence in percent, divide by 100.) To calculate prevalence, the IDEA autism counts were divided by total statewide public school age populations by grade, matching to the appropriate IDEA age (e.g, kindergartners = 5 year-olds, first graders = 6 year-olds, etc.) from the National Center for Education Statistics (NCES) (http://nces.ed.gov/ccd/elsi/). As noted in the main text, children in private schools are included in IDEA data but not in NCES data, such that the uncorrected ratio probably overestimates the actual prevalence. However, due to lack of sufficient information on private:public school population ratios for all 50 states dating back to 1991, Supplementary File S3 does not attempt to correct for the missing private school component of the denominator.

**S4 Dataset. ADDM autism prevalence for 15 states**

Supplementary File S4 presents autism prevalence data from the Autism and Developmental Disabilities Monitoring (ADDM) Network, which is a surveillance system established by the Centers for Disease Control (CDC) to provide estimates of autism prevalence among 8 year-old children. Reports are available every 2 years for birth years 1992-2004, for a total of 7 biannual reports. Additional data were obtained for birth year 1998 from New Jersey, which was out of the ADDM network for that cycle, but nonetheless completed ASD surveillance by the CDC-ADDM method [*Zahorodny et al.*, 2014]. ADDM cases are determined based on abstracts of pediatric health clinic and special education reports, which are then reviewed by trained clinicians to determine each child’s status. Over the lifetime of the network, ADDM has covered parts of 15 different states, but the states covered are not always consistent from report to report and the number of counties covered in each successive report is also somewhat variable. These differences, along with the ADDM prevalence in each of the 7 reports, are presented in Supplementary File S4. ADDM also computes an overall U.S. autism prevalence through a simple mean of the participating states.
